# The interleukin-1 receptor antagonist anakinra improves endothelial dysfunction in streptozotocin-induced diabetic rats

**DOI:** 10.1186/s12933-014-0158-z

**Published:** 2014-12-18

**Authors:** Susana Vallejo, Erika Palacios, Tania Romacho, Laura Villalobos, Concepción Peiró, Carlos F Sánchez-Ferrer

**Affiliations:** Departamento de Farmacología, Facultad de Medicina, Universidad Autónoma de Madrid, Calle Arzobispo Morcillo 4, 29029 Madrid, Spain; Present address: Departamento de Ciencias de la Salud, Edificio CN208, Oficina O, Universidad de las Américas, Puebla, México; Present address: Paul Langerhans-Group, Integrative Physiology, German Diabetes Center, Auf’m Hennekamp 65, 40225 Düsseldorf, Germany

**Keywords:** Diabetes mellitus, Endothelial dysfunction, NADPH oxidase, Nuclear factor-κB, Anakinra, Interleukin-1β, Vascular inflammation

## Abstract

**Background:**

Endothelial dysfunction is a crucial early phenomenon in vascular diseases linked to diabetes mellitus and associated to enhanced oxidative stress. There is increasing evidence about the role for pro-inflammatory cytokines, like interleukin-1β (IL-1β), in developing diabetic vasculopathy. We aimed to determine the possible involvement of this cytokine in the development of diabetic endothelial dysfunction, analysing whether anakinra, an antagonist of IL-1 receptors, could reduce this endothelial alteration by interfering with pro-oxidant and pro-inflammatory pathways into the vascular wall.

**Results:**

In control and two weeks evolution streptozotocin-induced diabetic rats, either untreated or receiving anakinra, vascular reactivity and NADPH oxidase activity were measured, respectively, in isolated rings and homogenates from mesenteric microvessels, while nuclear factor (NF)-κB activation was determined in aortas. Plasma levels of IL-1β and tumor necrosis factor (TNF)-α were measured by ELISA. In isolated mesenteric microvessels from control rats, two hours incubation with IL-1β (1 to 10 ng/mL) produced a concentration-dependent impairment of endothelium-dependent relaxations, which were mediated by enhanced NADPH oxidase activity via IL-1 receptors. In diabetic rats treated with anakinra (100 or 160 mg/Kg/day for 3 or 7 days before sacrifice) a partial improvement of diabetic endothelial dysfunction occurred, together with a reduction of vascular NADPH oxidase and NF-κB activation. Endothelial dysfunction in diabetic animals was also associated to higher activities of the pro-inflammatory enzymes cyclooxygenase (COX) and the inducible isoform of nitric oxide synthase (iNOS), which were markedly reduced after anakinra treatment. Circulating IL-1β and TNF-α levels did not change in diabetic rats, but they were lowered by anakinra treatment.

**Conclusions:**

In this short-term model of type 1 diabetes, endothelial dysfunction is associated to an IL-1 receptor-mediated activation of vascular NADPH oxidase and NF-κB, as well as to vascular inflammation. Moreover, endothelial dysfunction, vascular oxidative stress and inflammation were reduced after anakinra treatment. Whether this mechanism can be extrapolated to a chronic situation or whether it may apply to diabetic patients remain to be established. However, it may provide new insights to further investigate the therapeutic use of IL-1 receptor antagonists to obtain vascular benefits in patients with diabetes mellitus and/or atherosclerosis.

## Background

Vascular disease is a common feature in patients with diabetes mellitus, impaired glucose tolerance, and obesity. Diabetic vasculopathy is associated with endothelial dysfunction and impaired vascular reactivity through a mechanism that involves the over-production of reactive oxygen species, as observed in both humans and experimental animal models [1-9].

In addition to oxidative stress, other mechanisms are participating in diabetic vasculopathy, including insulin resistance and increased systemic and vascular inflammation [10]. In this context, increasing evidence supports the participation of pro-inflammatory cytokines in the development of vascular diseases, such as atherosclerosis and diabetic vasculopathy [11-14]. In particular, interleukin-1β (IL-1β) has been involved in the pathogenesis of type 1 and type 2 diabetes [15,16], as well as in the development of the diabetic retinopathy associated to type 1 diabetes [17,18]. Accordingly, the antagonism of IL-1 receptors has been recently proposed as a pharmacological target for the treatment of type 2 diabetes [19-21].

Despite all these observations, few data are available about the possible involvement of pro-inflammatory cytokines, and particularly IL-1β, in diabetic-induced endothelial dysfunction, although the ability of this cytokine to generate an impairment of endothelium-dependent relaxations has been previously reported in mesenteric arteries from non-diabetic rats [22-24]. In the present work, we tested the hypothesis that vascular inflammation mediated by cytokines, such as IL-1β, may play an important role in the development of diabetic endothelial dysfunction. Using a short-term model of streptozotocin-induced diabetic rat, we found that anakinra, a recombinant human antagonist of IL-1 receptors [25] improves the endothelial dysfunction observed in the isolated mesenteric microvasculature. Additionally, we found that such effects of anakinra were likely mediated by interfering with the pro-oxidant and pro-inflammatory mechanisms triggered by diabetes into the vascular wall.

## Methods

### Ethical approval

All animal studies were performed according to national and European guidelines (2010/63/EU), approved by the ethics committee of Universidad Autónoma de Madrid (CEI 27–670), and developed in registered animal facilities (ES-28079-000097).

### Experimental animals

**Figure 1:**
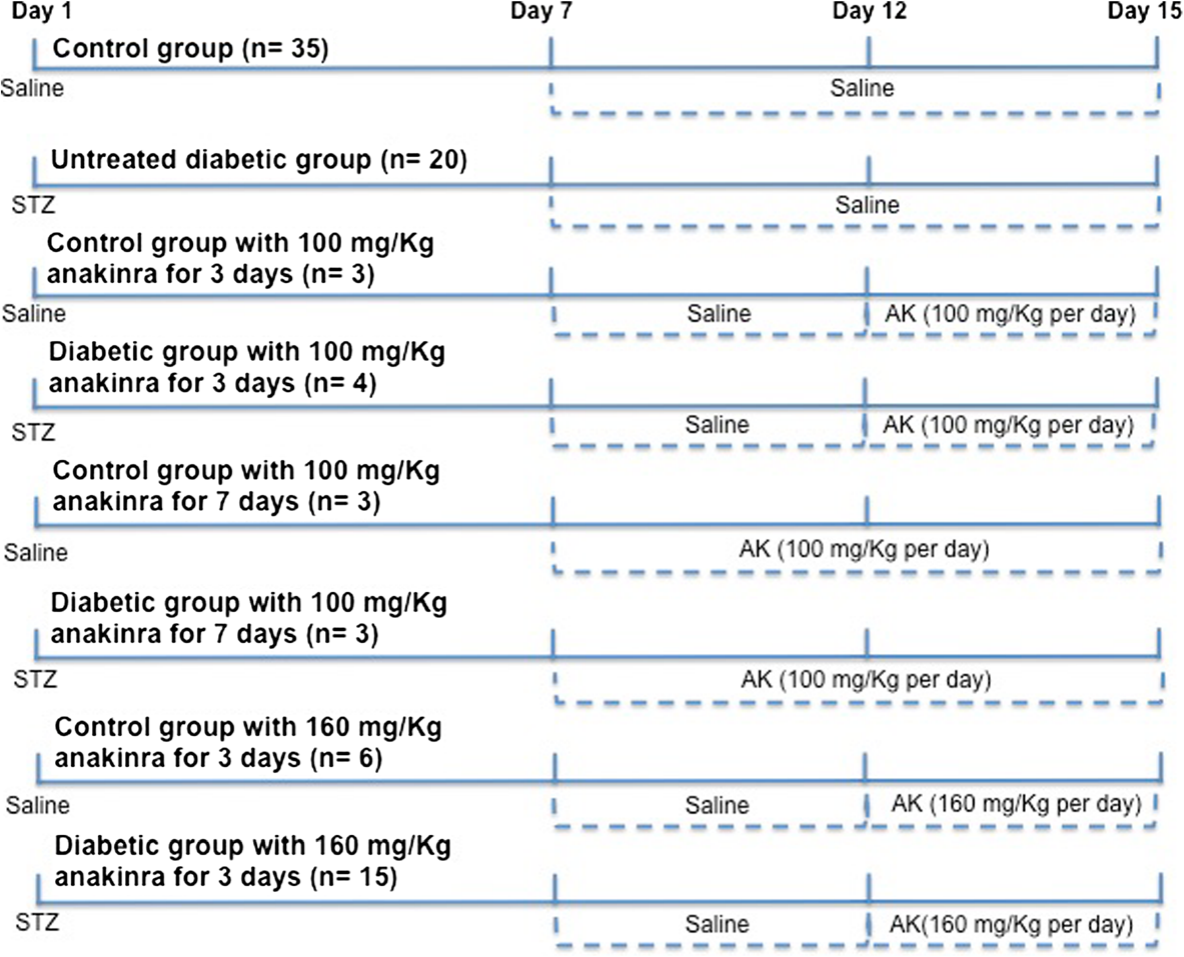
**Diagram for animal treatments.** Diagram of the different designed rat groups, both in control conditions or after diabetes mellitus induced by i.p. administration of streptozotocin (STZ). The animals received further i.p. administration of saline and/or anakinra (AK) before sacrifice in day 15.

In 16-week male old Sprague–Dawley (SD) rats (300 to 350 g), insulin-dependent diabetes was induced by a single administration of streptozotocin (60 mg kg^−1^; i.p.). After 72 hours, tail blood samples were obtained and glucose concentration was measured using a glucometer Optium Xceed (MediSense, Abbott Laboratories, Chicago, IL, USA). Diabetes induction was considered successful when glycaemia was higher than 20 mmol/L. Control age- and mass-matched rats were injected with saline solution and kept in similar conditions as diabetic animals. All the animals had *ad libitum* access to food (standard rat chow diet) and tap water. The experimental animals were separated into eight groups schematized in Figure 1.

### Drug effects on vascular tone of mesenteric microvessels

The animals were briefly exposed to a chamber filled with carbon dioxide until they fell unconscious and then immediately killed by cervical dislocation. The mesentery was removed, and placed in a Petri dish containing Krebs-Henseleit solution (KHS) at 4°C. The third branch mesenteric arteries were dissected in control and diabetic rats (mean internal diameter ranged between 200 and 400 μm; non-significant differences were observed among the different groups of rats). The arteries were dissected and cleaned free of fat and connective tissue under a light microscope and mounted as ring preparations on a small vessel myograph [6] to measure isometric tension. Arteries were bathed in KHS at 37°C continuously bubbled with a 95% O_2_-5% CO_2_ mixture, which yields a pH of 7.4 and their passive tension and internal circumference were determined. The arteries were subjected to optimal tension (90% of the tension equivalent to a intramural pressure of 100 mm Hg. After 30 min of equilibration, the vessels were exposed to 125 mmol/L KCL (KKHS, equimolar substitution of KCl for NaCl in KHS) for 2 min in order to check their functional integrity. Segments failing to achieve a maximum active tension equivalent to a pressure of 100 mmHg were rejected [6].

The bath was then washed three times with KHS and a further 150 min washout period was allowed before the arteries were contracted with the concentration of NA (1 μmol/L) required to produce approximately 80% of the maximum response to KKHS. Relaxations to ACh were subsequently assessed by adding cumulative concentrations of the drug at 2 min intervals (final bath concentrations 0.1 nmol/L to 10 μmol/L). Some microvessels were pre-incubated with increasing concentrations of IL-1β (1, 2.5, 5, or 10 ng/mL) 120 min before and during the administration of NA and ACh. Other vascular segments were treated with the IL-1 receptors antagonist anakinra (10, 50, or 100 μg/mL), the inhibitor of NADPH oxidase activity apocynin (10 μmol/L), the anion superoxide scavenger tempol (100 μmol/L), the cyclooxygenase (COX) inhibitor indomethacin (10 μmol/L), or the blocker of the inducible isoform of nitric oxide synthase (iNOS) 1400 W (10 μmol/L), for 30 min in advance and during the administration of IL-1β, NA, and ACh, respectively. Finally, in selected experiments, instead of ACh, concentration-dependent curves to the endothelium-independent vasodilator sodium nitroprusside (SNP; 1 nmol/L to 10 μmol/L) were performed.

### NADPH oxidase activity assay

The activity of NADPH oxidase was measured in rat microvascular preparations by lucigenin-derived chemiluminiscence, as previously described [26]. In these experiments, the mesenteric arteries were dissected, as previously indicated and homogeneously distributed into tubes containing 1 mL KHS at 37°C continuously bubbled with a 95% O_2_-5% CO_2_ mixture. When appropriate, the microvessels were treated for 30 min with 2.5 ng/mL IL-1β, in the absence or in the presence of anakinra (100 μg/mL) or apocynin (10 μmol/L). Afterwards, the microvascular preparations were homogenized in lysis buffer (pH 7.0) containing 50 mmol/L KH2PO4, 1 mmol/L EGTA and 150 mmol/L sucrose at 4°C. For every sample, the protein content was determined by the bicinchoninic acid method. Microvascular extracts were then incubated in phosphate-buffered saline containing 5 mmol/L lucigenin with or without 100 mmol/L NADPH. Luminescence was then measured every 10 seconds for 5 minutes in a microplate luminometer (Orion II Microplate Luminometer, Berthold Technologies, Bad Wildbad Germany). For every sample, NADPH oxidase activity was calculated as the difference between the activities obtained in the presence and in the absence of NADPH. The enzymatic activity was expressed as relative light units (RLU)/mg of protein/min.

### In situ detection of activated NF-κB

Southwestern histochemistry is a non-radiactive technique developed for *in situ* detection of activated transcription factors, as described in detail previously [27]. This method requires the use of a double-strand DNA containing the consensus sequence of NF-κB. In brief, NF-κB consensus oligonucleotides were digoxigenin labeled with a 3’-terminal transferase. Paraffin-embedded tissue sections were fixed in 0.5% paraformaldehyde and subsequently digested with 0.5% pepsin and 0.1 mg/mL DNaseI. Samples were then incubated at 37°C with 12 pmol/L of digoxigenin-labeled NF-κB probe in buffer containing 0.25% BSA and 2 μg/mL poly(dI-dC), followed by alkaline phosphatase-conjugated anti-digoxigenin IgG and colorimetric detection with nitro blue tetrazolium/5-bromo-4-chloro-3-indolyl phosphate toluidine salt (NBT/BCIP). The nuclear staining in blue gives evidence for activated transcription factor. To verify the specificity of the Southwestern histochemistry, three different controls were systematically performed [28]: (1) Competition assay: incubation with a 200-excess of unlabeled probe reduced the intensity of the nuclear staining; (2) Mutant labeled probe: serial slides were incubated under the same conditions with consensus NF-κB probe or a mutant probe (with a modification in the NF-κB binding site). A very slight intensity was observed with mutant probe, as compared with consensus probe, demonstrating the specificity of the DNA binding; and (3) Negative control: no staining was observed in the absence of labeled probe.

### Cytokine determinations

At the time of the experiment, blood plasma samples were collected and stored at −80°C until assay were performed. Plasmatic IL-1β and tumour necrosis factor (TNF)-α levels were determined by ELISA (ELx800 Bio-Tek Instruments, Winoski, VT, USA) using an ELISA commercial kit (Gen Probe/Diaclone, San Diego, CA, USA).

### Materials

The composition of KHS (mmol/L) was NaCl 115, CaCl_2_ 2.5, KCl 4.6, KH_2_PO_4_ 1.2, MgSO_4_^**.**^7H_2_O 1.2, NaHCO_3_ 25, glucose 5.5 and Na_2_EDTA 0.03. Recombinant IL1β was obtained from PeproTech (PeproTech GmbH, Hamburg, Germany) while anakinra (*Kineret©*) was obtained from Biovitrum (Swedish Orphan Biovitrum AB, Stockholm, Sweden). Streptozotocin, NA, KCl, ACh, SNP, indomethacin, 1400 W, apocynin, tempol, lucigenin, and, unless otherwise stated, all other reagents were purchased from Sigma Chemical Co. (St. Louis, MO, USA). Noradrenaline was prepared in saline (0.9% NaCl)-ascorbic acid (0.01% w/v). Streptozotocin was dissolved in citric acid-trisodium citrate (0.1 mmol/L) buffer with a pH of 4.5. Lucigenin and NADPH were dissolved in saline solution with 0.1 mmol/L EDTA, and 10 mmol/L Tris/HCl, respectively. All other drug solutions were made in distilled water.

### Statistical analysis

Results are expressed as mean ± standard error (SEM). pEC50 values for ACh were defined as the negative log of the effective concentrations required to produce half the maximum effect. In the figures, n means the number of vascular segments used. Statistical analysis was performed using two-factor ANOVA for curves or Student’s *t*-test for data points, with the level of significance chosen at p < 0.05.

## Results

### Effects of IL-1β on endothelium-dependent relaxations

**Figure 2:**
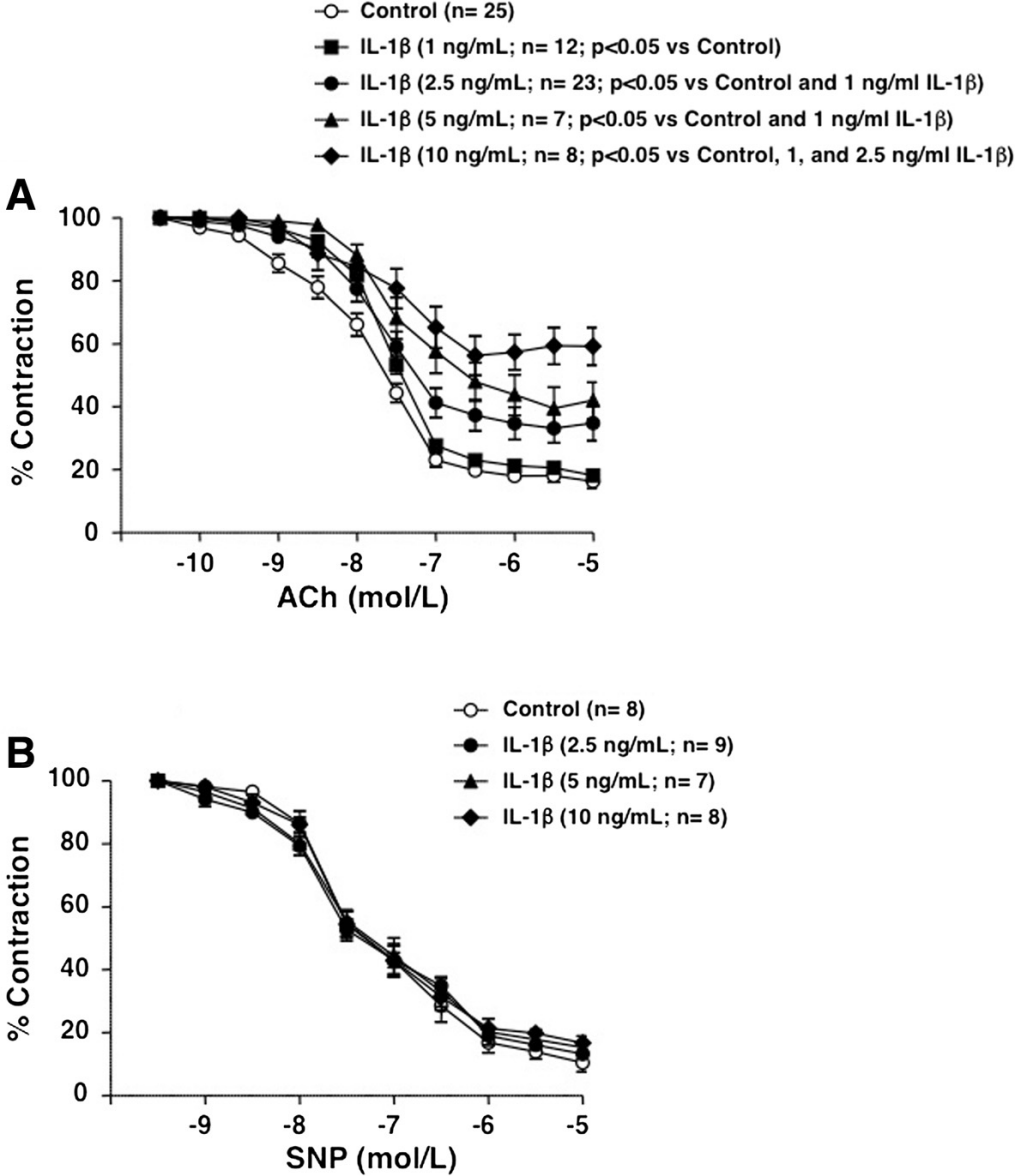
**IL-1β impairs vascular reactivity. (A)** Effect of IL-1β **(**1, 2.5, 5, and 10 ng/mL) on the endothelium-dependent relaxations induced by acetylcholine (ACh, 0.1 nmol/L to 10 μmol/L) in isolated mesenteric arteries from control Sprague–Dawley (SD) rats. Data are expressed (means ± SE) as the percentage of the previous contraction induced with 1 μmol/L noradrenaline (NA). The number of segments used for every curve, which were obtained from 5 to 12 animals, as well as the statistical significance are in parenthesis. **(B)** Effect of IL1β (2.5, 5, and 10 ng/mL) on the endothelium-independent relaxations induced by sodium nitroprusside (SNP, 1 nmol/L to 10 μmol/L) in isolated mesenteric arteries from control SD rats. Data are expressed as the percentage of the pre-contraction induced with 1 μmol/L NA. The number of segments used for every curve, which were obtained from 4 animals, as well as the statistical significance are in parenthesis.

When isolated mesenteric microvessels from SD rats were incubated for 2 hours with IL-1β (1 to 10 ng/mL), there were no changes in the contractile responses to 1 μmol/L NA (10.19 ± 0.90, 12.13 ± 0.81, 9.09 ± 1.24, 11.85 ± 1.21, and 10.51 ± 3.56 mNewtons, for control and segments treated with 1, 2.5, 5, and 10 ng/mL IL-1β, respectively), while a concentration-dependent impairment of the endothelium-derived relaxations induced by ACh was observed (Figure 2A). On the other hand, IL-1β (2.5, 5, and 10 ng/mL IL-1β, respectively) did not affect the endothelium-independent relaxations evoked by SNP (Figure 2B). A submaximal concentration of 2.5 ng/mL IL-1β was used in the next experiments.

**Figure 3:**
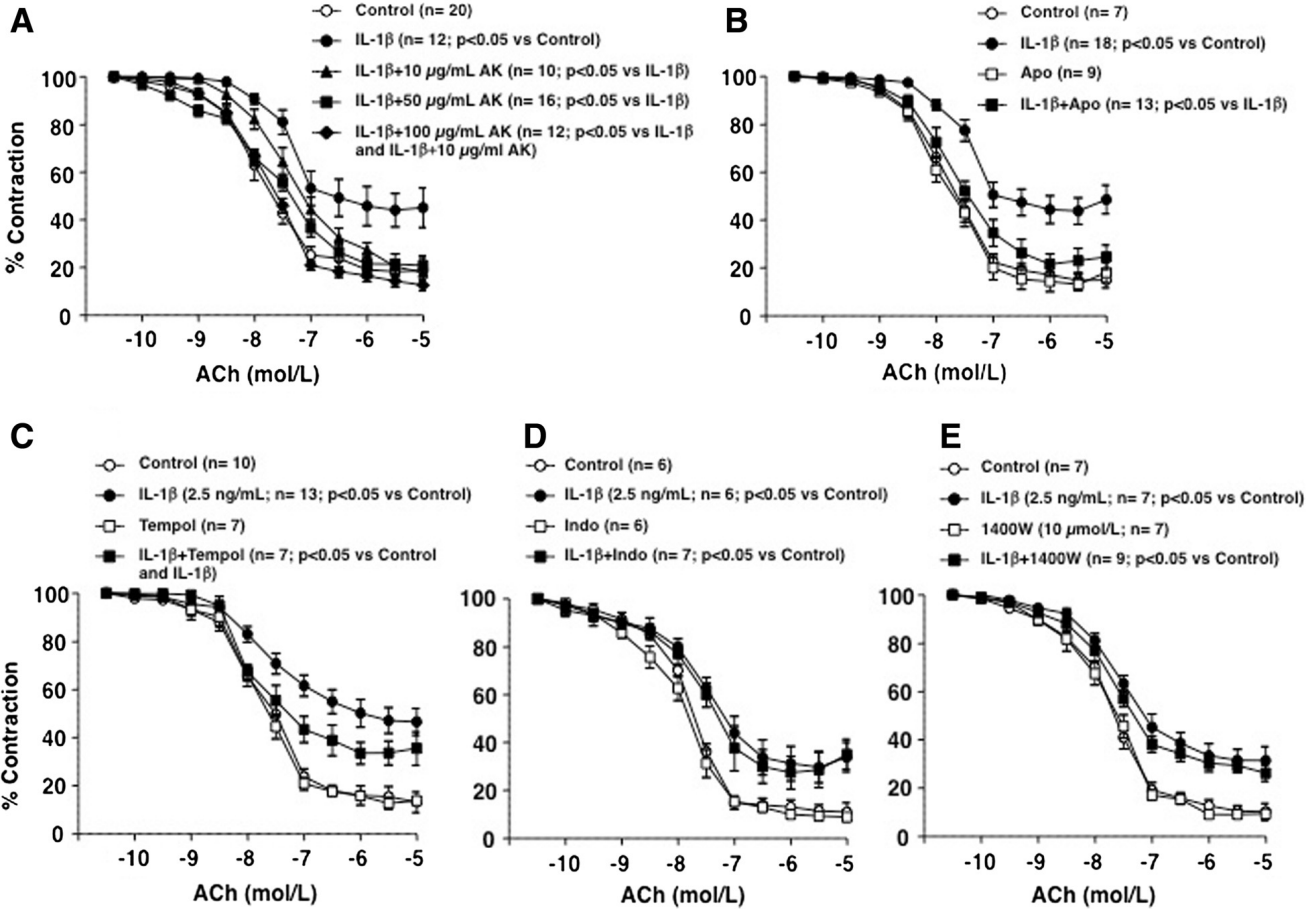
**Mechanisms involved in the endothelium impairment by IL-1β.** Effects of **(A)** anakinra (AK, 10, 50, and 100 μg/mL), **(B)** apocynin (Apo; 10 μmol/L), **(C)** tempol (100 μmol/L), **(D)** indomethacin (Indo; 10 μmol/L), or **(E)** 1400 W (10 μmol/L) on the impairment produced by 2.5 ng/mL IL-1β on the endothelium-dependent relaxations induced by ACh (0.1 nmol/L to 10 μmol/L) in isolated mesenteric arteries from control SD rats. Data are expressed (means ± SE) as the percentage of the previous contraction induced with 1 μmol/L NA. The number of segments used for every curve, which were obtained from 3 to 14 animals, as well as the statistical significance are in parenthesis.

The impairment of endothelium-dependent relaxations evoked by IL-1β was antagonized by the competitive inhibitor of IL-1 receptors anakinra (10, 50, and 100 μg/mL) in a concentration-dependent manner (Figure 3A). Anakinra neither affected the ACh-evoked vasodilatations in the absence of IL-1β (data not shown) nor the NA-induced contractility (8.94 ± 0.87, 9.10 ± 0.99, 12.07 ± 1.33, 10.98 ± 0.75, and 9.86 ± 1.56 mNewtons, in control and 10, 50, or 100 μg/mL AK, respectively). The endothelium impairment induced by 2.5 ng/mL IL-1β was blunted by pre-incubating the vascular segments with the NADPH oxidase inhibitor apocynin (10 μmol/L) (Figure 3B) and partially prevented by the anion superoxide scavenger tempol (100 μmol/L) (Figure 3C). On the other hand, no role for pro-inflammatory enzymes, such as COX or iNOS, was observed for the endothelial dysfunction evoked by IL-1β, as it was not modified by the inhibition of COX with indomethacin (10 μmol/L) (Figure 3D) or by the blockade of iNOS with 1400 W (10 μmol/L) (Figure 3E). None of these treatments did modify the contractile responses evoked by 1 μmol/L NA (data not shown).

### Effects of IL-1β on microvascular NADPH oxidase activity

**Figure 4:**
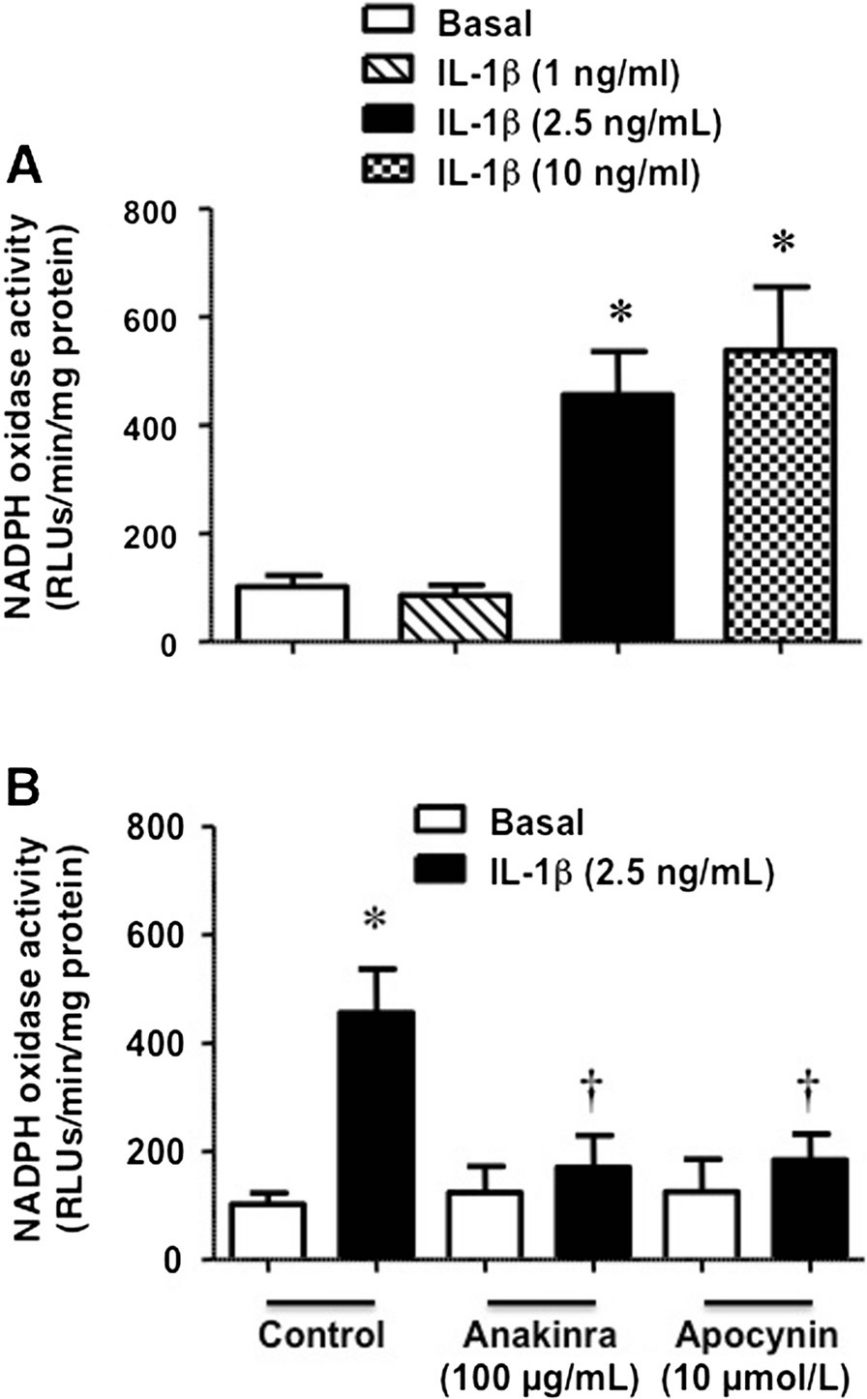
**IL-1β increases vascular NADPH oxidase activity. (A)** NADPH oxidase activity in microvascular mesenteric preparations from control SD rats after 30 min stimulation with IL-1β (1, 2.5, and 10 μg/mL). Bars represents the mean ± SE of the relative light units (RLU) obtained in 3 different experiments using tissues from 6 animals. **(B)** NADPH oxidase activity in microvascular mesenteric preparations from control SD rats after 30 min stimulation with 2.5 μg/mL IL-1β, either alone and in the presence of 100 μg/mL anakinra or 10 μmol/L apocynin. Bars represent the mean ± SE of the relative light units (RLU) obtained in 3 experiments using tissues from 9 animals. *p < 0.05 vs basal; †p < 0.05 vs IL-1β.

After treating isolated mesenteric arteries from control SD rats with IL-1β (1, 2.5, and 10 ng/mL), a concentration-dependent enhancement of the NADPH oxidase activity was observed in the microvascular preparations (Figure 4A). The enhancement of the enzymatic activation evoked by a submaximal concentration of 2.5 ng/mL IL-1β was abolished by both anakinra (100 μg/mL) and apocynin (10 μmol/L; Figure 4B).

### Effects of *in vivo* treatment with anakinra on diabetic endothelial dysfunction

**Figure 5:**
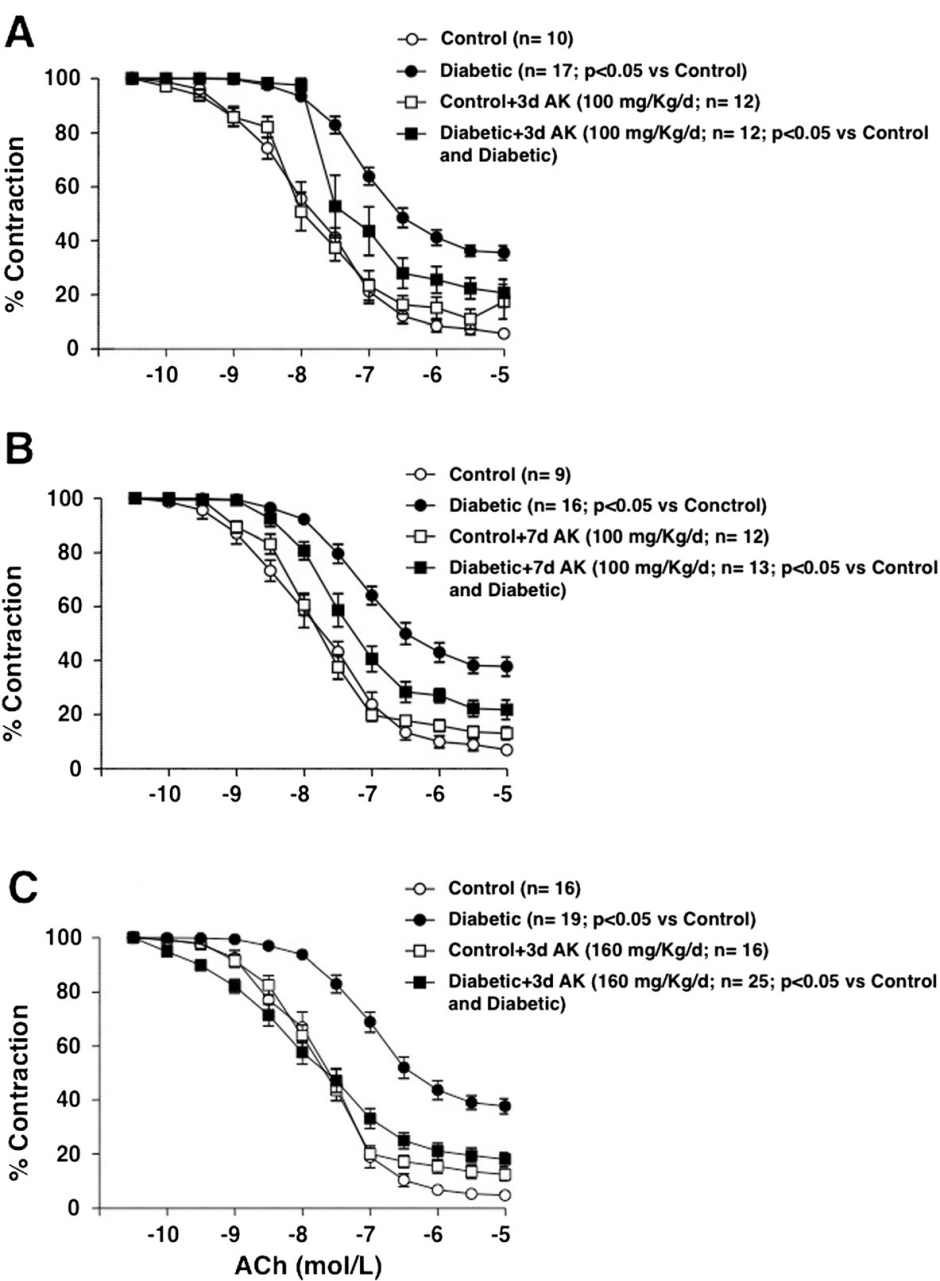
**Anakinra improves endothelial function in diabetic rats.** Effects of treatment with anakinra (AK) (**A**; 100 mg/Kg/day for 3 days), (**B**; 100 mg/Kg/day for 7 days), and (**C**; 160 mg/Kg/day for 3 days) on the endothelium-dependent relaxations to ACh (0.1 nmol/L to 10 μmol/L) in isolated mesenteric arteries from control and two weeks evolution streptozotocin-induced diabetic SD rats. Data are expressed (means ± SE) as the percentage of the previous contraction induced with 1 μmol/L NA. The number of segments used for every curve, which were obtained from 4 to 10 animals, as well as the statistical significance are in parenthesis.

In accordance to our previous report [7], two weeks of diabetes evolution after streptozotocin administration were required to develop significant endothelial dysfunction in the mesenteric microvessels isolated from diabetic rats. However, no modifications were observed in the contractile responses to 1 μmol/L NA (10.39 ± 0.75 and 10.30 ± 0.96 mNewtons for control and diabetic rats, respectively), nor in the endothelium-independent relaxations to SNP (data not shown). When the diabetic rats received 100 mg/Kg/day anakinra during three days prior to sacrifice, a significant improvement of the diabetic endothelial dysfunction was observed, despite a residual endothelial impairment (Figure 5A). Such recovery of endothelial function was not further ameliorated by expanding the treatment with anakinra for 7 days (Figure 5B). The administration of a higher dose of anakinra (160 mg/Kg/day for 3 days) produced a small but significant additional improvement of the ACh-induced dependent relaxations when compared with 100 mg/Kg/day anakinra for 7 days (p < 0.05), although a residual endothelial dysfunction still persisted (Figure 5C). None of the doses of anakinra used did modify endothelium-dependent relaxations in vessels from non-diabetic animals (Figures 5A, 5B, and 5C) or the contractile responses evoked by 1 μmol/L NA in control and diabetic animals (data not shown).

### NADPH oxidase activity and NF-κB activation in the vascular wall from diabetic rats

**Figure 6:**
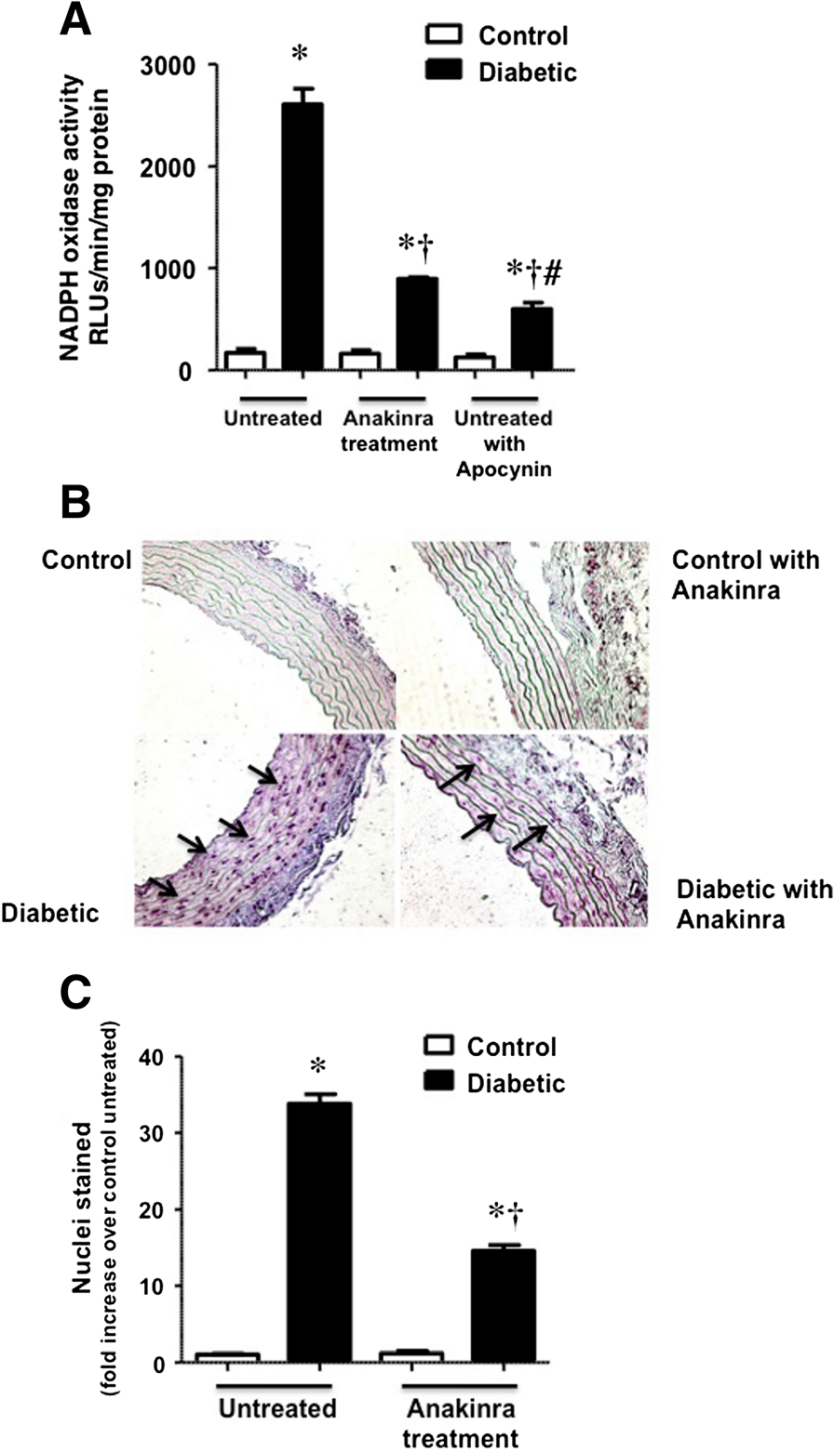
**Anakinra reduces vascular NADPH oxidase activity and inflammation in diabetic rats. (A)** NADPH oxidase activity in microvascular mesenteric preparations from control and two weeks evolution streptozotocin-induced diabetic SD rats, either untreated or treated for 3 days with anakinra (AK; 160 mg/Kg/day). Some samples also received, before determination, 10 μmol/L apocynin. Bars represent the mean ± SE of the relative light units (RLU) obtained in at least 3 experiments using tissues from 12 different animals. **(B)** Representative photomicrographs (20X) of microscopic sections of control and diabetic rat aorta, either untreated or receiving AK (160 mg/Kg/day, 3 days) hybridized with an oligonucleotide with the NF-κB consensus site. Preparations without probe were used as negative controls and a 200-fold excess of unlabeled probe was used to test the specificity of the technique. Arrows indicate stained nuclei. **(C)** Quantitative analysis of the number of NF-κB stained nuclei by squared micrometers in the aorta from control and diabetic rats, either untreated or receiving AK. Bars (mean ± SE) represent the fold increase over untreated control stained nuclei, which averaged 0.73 ± 0.09/10,000 μm^2^. At least 3 different animals were employed in every case. *p < 0.05 vs non-diabetic control rats; †p < 0.05 vs diabetic rats; #p < 0.05 vs AK-treated diabetic rats.

In mesenteric microvascular preparations obtained from diabetic rats after two weeks of evolution, there was a significant increase in NADPH oxidase activity, which was significantly reduced when the isolated vessels were treated 30 min before determination with 10 μmol/L apocynin (Figure 6A). When the mesenteric microvessels were obtained from diabetic animals receiving anakinra (160 mg/Kg/day for 3 days), a marked reduction in NADPH-oxidase enzymatic activity was also observed (Figure 6A).

NF-κB activation was localized by Southwestern histochemistry in aortas from control and diabetic rats untreated or receiving 160 mg/Kg day for 3 days. The arterial sections were hybridized with an oligonucleotide containing the consensus sequence of the NF-κB recognition site. As shown in Figure 6B, the sections from control animals, treated or not with anakinra, did not show any staining for NF-κB. On the contrary, sections from diabetic rats showed a significant increase in nuclei activation, which was reduced by treatment with anakinra (Figure 6B). The quantification of activated nuclei is depicted in Figure 6C.

### Role for oxidative stress and inflammation into the vascular wall on diabetic endothelial dysfunction

**Figure 7:**
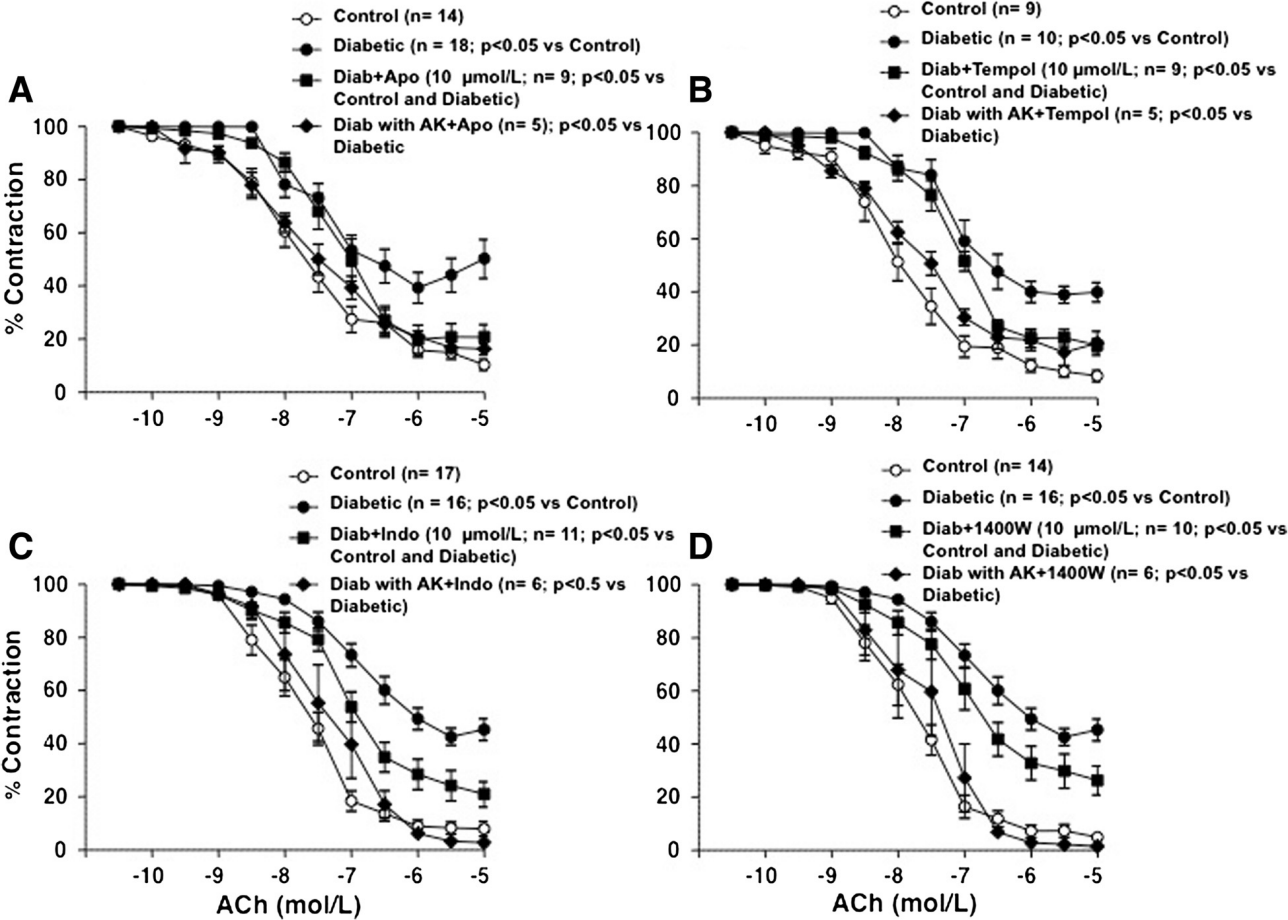
**Anakinra attenuates the vascular pro-oxidant and pro-inflammatory environment leading to diabetic endothelial dysfunction.** Effect of **(A)** tempol (100 μmol/L), **(B)** apocynin (Apo, 10 μmol/L), **(C)** indomethacin (Indo; 10 μmol/L), or **(D)** 1400 W (10 μmol/L) on the impairment of the endothelium-dependent relaxations evoked by ACh (0.1 nmol/L to 10 μmol/L) observed in diabetic SD rats, either untreated or receiving anakinra (AK, 160 mg/Kg/dasy during 3 days). Data are expressed as the percentage of the pre-contraction induced with 1 μmol/L NA. The number of segments used for every curve, which were obtained from 3 to 5 animals, as well as the statistical significance are in parenthesis.

The endothelial dysfunction induced in mesenteric microvessels by two weeks evolution diabetes involved the activation of vascular pro-oxidant and pro-inflammatory pathways, since the dysfunction was partially prevented by pre-incubation with the NADPH oxidase blocker apocynin (Figure 7A), the superoxide anion scavenger tempol (Figure 7B), the COX blocker indomethacin (Figure 7C), or the iNOS inhibitor 1400 W (Figure 7D). In microvessels from diabetic rats treated with anakinra (160 mg/Kg/day for 3 days), and further incubated in the presence of apocynin, tempol, indomethacin, or 1400 W, the ACh-induced relaxations were similar to those obtained in vessels from control animals, without any remaining endothelial dysfunction (Figures 7A, 7B, 7C, and 7D). None of the drugs used modified endothelium-dependent relaxations in vessels from non-diabetic animals (Figures 3B, 3C, 3D, and 3E), or the contractile responses evoked by 1 μmol/L NA in control or diabetic animals (data not shown).

### Plasma IL-1β and TNF-α determinations

The pro-inflammatory cytokines IL-1β and TNF-α were determined by ELISA in plasmatic samples from control diabetic rats, either without treatment or after receiving anakinra (100 mg/Kg/day for 3 days; 100 mg/Kg/days for 7 days, or 160 mg/Kg/day for 3 days). As compared with control animals, no significant changes in cytokine levels were observed in untreated diabetic animals neither in control animals treated with anakinra (Table 1). However, anakinra treatment (100 mg/Kg/day for 7 days and 160 mg/Kg/day for 3 days) reduced significantly the plasmatic levels of IL-1β and TNF-α in diabetic animals (Table 1).

**Table 1:** **Plasmatic levels of IL-1β and TNF-α (pg/mL) in control and two weeks evolution streptozotocin-induced diabetic SD rats, both untreated or receiving i.p. anakinra (AK)**

		**Untreated**	**AK (100 mg/Kg/day for 3 days)**	**AK (100 mg/Kg/day for 7 days)**	**AK (160 mg/Kg/day for 3 days)**
**Control**	**IL-1β**	82.61 ± 5.02	78.59 ± 18.02	77.85 ± 18.55	90.44 ± 18.47
	**TNF-α**	11.52 ± 2.71	12.82 ± 3.08	12.39 ± 1.30	10.21 ± 4.34
**Diabetic**	**IL-1β**	83.88 ± 10.84	57.11 ± 5.75	45.17 ± 8.48*	42.89 ± 4.92*†
	**TNF-α**	11.22 ± 2.05	8.26 ± 2.13	3.48 ± 0.82*†	3.43 ± 1.27*†

Data are expressed as mean ± SE of 4 to 10 determinations. *p < 0.05 vs respective untreated rats; †p < 0.05 vs respective control group.

## Discussion

It is well known that the development of an inflammatory environment in the vasculature is followed by a clear impairment of endothelial function; indeed, acute systemic inflammation with *Salmonella typhi* vaccine produces a temporary but profound dysfunction of human arterial endothelium in both resistance and conduit vessels, which is related to cytokine production [29,30]. On the other hand, pro-inflammatory cytokines, such as TNF-α, induce endothelial dysfunction in animal models and humans [31-33]. Moreover, there is increasing evidence of a role for pro-inflammatory cytokines in the development of vascular diseases, particularly diabetic vasculopathy [11-15]. In this line, it has been proposed that TNF-α can be one of the mediators for the insulin resistance and endothelial dysfunction associated to type 2 diabetes mellitus [33-35].

Endothelial dysfunction produced by IL-1β has been previously reported in isolated rat mesenteric microvessels using both short (30 min) [22] and longer (14 h) [24] incubation times with the cytokine. We selected a 2 h period of IL-1β treatment as it produced a selective concentration-dependent endothelial dysfunction, without affecting the contractile or relaxant responses of the vascular smooth muscle layer. Moreover, the pro-inflammatory responses triggered by IL-1β were not entirely developed after this short incubation time with the cytokine, as indicated by the lack of activity of pro-inflammatory enzymes such as COX and iNOS. Therefore, in the present conditions, we assume that the impairment of the endothelium-dependent relaxations evoked by IL-1β in isolated segments from non-diabetic animals was mainly mediated by an enhancement of superoxide anion production produced by the activation of NADPH oxidase. Endothelial alteration by IL-1β was requiring the activation of IL-1 receptors, since the pre-incubation of the vascular segments with the recombinant human antagonist of IL-1 receptors anakinra led to a concentration-dependent recovery of the endothelial dysfunction evoked by IL-1β. In addition, anakinra also blocked NADPH oxidase activation by IL-1β to the same extent than apocynin, further supporting the role for NADPH oxidase-derived superoxide anions as mediators of the cytokine-induced endothelial dysfunction.

Increased production of IL-1β has been linked to a pro-inflammatory environment in the pancreatic islets that leads to beta-cell dysfunction and death in type 2 diabetes mellitus [36,37]. It has been also reported that IL-1β may have a role in the pathogenesis of diabetic retinopathy [17]. Furthermore, an enhanced expression of IL-1β in high glucose conditions has been described in human monocytes and macrophages [38-40], human pancreatic islets [36], and human aortic endothelium [41], while the up-regulation of IL-1β has been described in the retina and retinal vessels from diabetic rats [19]. Moreover, lower adipose tissue inflammation occurs in knock-out mice for interleukin-1 receptor-I receiving a high fat-diet, although these animals present similar immune cell recruitment than controls [42].

Based on these studies, the human recombinant antagonist of IL-1 receptors anakinra was tested in patients with type 2 diabetes mellitus, showing an improvement of glycaemia and beta cell secretory function, as well as a reduction of systemic markers of inflammation [19], which were maintained for several months after treatment withdrawal [43]. Furthermore, studies in experimental models indicate that IL-1 receptor antagonists reduce hyperglycaemia and inflammation in type 2 Goto-Kakizaki diabetic rats [44,45], and mice with diet-induced hyperglycaemia or obesity [46,47]. Moreover, anakinra improves cholesterol and adiponectin levels levels in *db-/db-* diabetic mice, reducing the ischemia-induced endoplasmic reticulum stress and inflammation [48].

On the other hand, the relevance for interleukin-1 receptor signalling on the development of atherosclerosis has been clearly demonstrated in knock-out mice models for these receptors [49-51]. Interestingly, treatment with anakinra ameliorates the cardiac remodelling process in experimental acute myocardial infarction in rats [52], while in humans anakinra reduces the inflammatory responses observed in patients with heart failure after myocardial infarction [53,54]. Moreover, anakinra induces a recovery of the endothelial dysfunction in patients with rheumatoid arthritis [55]. Therefore, at present, interleukin-1 receptor inhibition is offering an interesting anti-inflammatory approach for treating not only diabetic patients, but also cardiovascular diseases, such as atherosclerosis [56].

The experiments with exogenous IL-1β were consistent with our hypothesis about the possible role for this cytokine as mediator of diabetic endothelial dysfunction. Therefore, we next aimed to determine whether this early endothelial alteration during diabetes, which occurs both in experimental models and patients and is closely related with enhanced oxidative stress [1-9], might be linked to mechanisms triggered by IL-1β and recovered by a competitive antagonist of IL-1 receptors, such as anakinra. As a recent-onset model of diabetes mellitus presenting endothelial dysfunction, we used streptozotocin-induced diabetic rats with two weeks of evolution, which is the earliest time required to observe endothelial dysfunction in this experimental model [7]. This point was first checked in the present study, since other diabetic models, like the Zucker diabetic fatty rat, can present quite well preserved endothelium-dependent relaxations in these early phases of the disease [57]. Also, as previously reported in our laboratory [5-8], we observed a recovery of the diabetes-induced endothelial dysfunction by scavenging superoxide anions, which indicates a crucial role for oxidative stress. Moreover, in agreement with data previously reported by others [58,59], our results clearly suggested that both the enhancement of oxidative stress and the endothelial dysfunction in this diabetic model were related to increased NADPH oxidase activity, since both alterations were similarly antagonised by the NADPH oxidase inhibitor apocynin. Our results also indicate an important activation of the inflammatory pathways into the vascular wall, linked to diabetes mellitus, based on the marked increase for NF-κB activation, together with the relevant participation for pro-inflammatory enzymes, like COX and iNOS, in the mechanisms producing endothelial dysfunction.

Being an early indicator for cardiovascular disease, prevention of the diabetic endothelial dysfunction is arising as an important therapeutic target that may not be reached with current antidiabetic treatments [60]. Interestingly, the acute treatment for 3 days of the streptozotocin-induced diabetic rats with 100 or 160 mg/Kg/day anakinra, which are at the higher dose range reported in experimental models [52,61], resulted in significant improvements of endothelium-dependent relaxations that seemed to be closely associated to the normalization of the NADPH oxidase activity in the vascular wall, as well as to a significant reduction in the vascular inflammatory responses. To our knowledge, this is the first report demonstrating that anakinra improves diabetic endothelial dysfunction. Furthermore, our results strongly suggest that the mechanism for such effect involves the antagonism of IL-1 receptors, which interferes with the IL-1β-evoked NADPH oxidase activity, leading to a lower NF-κB activation and reducing the enhanced activity of COX and iNOS. It is worth to note that the dose of 160 mg/Kg/day of anakinra produces effects only slightly higher than 100 mg/Kg/day, while the improvement of the endothelial dysfunction by 100 mg/Kg/day anakinra did not increase after prolonging the treatment up to 7 days. This is likely because we were using maximal or submaximal concentrations of the IL-1 receptor antagonist. Therefore, any possible extrapolation to patients should consider the effects of longer administration of much lower doses of anakinra.

We next aimed to study a possible role for enhanced circulating IL-1β in the observed diabetic endothelial dysfunction, determining its plasmatic levels in the streptozotocin-induced diabetic rats, as well as those of other pro-inflammatory cytokines, like TNF-α. However, despite the results obtained with anakinra, we could not detect any increase in the circulating concentrations of both cytokines after two weeks evolution of diabetes. This is in agreement with previous results determining IL-1β in this model [62], although increased serum levels of TNF-α have been detected in high fat fed/streptozotocin-induced type 2 diabetic rats [63]. Indeed, an enhancement in the plasmatic IL-1β or TNF-α concentrations is even hardly detected in diabetic patients [64-66]. Therefore, it seems reasonable to suggest that an early tissular inflammatory response into the vascular wall can be responsible, at least in part, for the diabetic endothelial dysfunction. Consistently, enhanced tissular inflammatory mediators have been described in vitreous samples from patients with proliferative diabetic retinopathy [67], as well as in kidney and liver from streptozotocin-induce diabetic rats [68,69].

## Conclusions

The present results indicates that the development of endothelial dysfunction in a short-term model of experimental type 1 diabetes can be related to an IL-1 receptor-mediated activation of vascular NADPH oxidase, leading to a subsequent enhancement of pro-oxidant and pro-inflammatory pathways into the vascular wall. Whether this mechanism can be extrapolated to a chronic situation or whether it may be applied to diabetic patients remains to be established. However, it may provide new insights to further investigate the therapeutic use of IL-1-receptor antagonists to obtain vascular benefits in patients with diabetes mellitus and/or atherosclerosis.

## Abbreviations

ACh: Acetylcholine
AK: Anakinra
ANOVA: Analysis of variance
Apo: Apocynin
COX: Cyclooxigenase
Indo: Indomethacin
IL-1β: Interleukin-1β
iNOS: Inducible nitric oxide synthase
KHS: Krebs-Henseleit solution
KKHS: Equimolar substitution of KCl for NaCl in KHS
NA: Noradrenaline
NADPH: Nicotinamide adenine dinucleotide phosphate
NF-κB: Nuclear factor-κB
SD: Sprague–Dawley rats
SEM: Standard error of the mean
SNP: Sodium nitroprusside
TNF-α: Tumor necrosis factor-α
